# Supplementary material for: Cooperativity of catalytic and lectin-like domain of Trypanosoma congolense trans-sialidase modulates its catalytic activity
Source: PLoS Negl Trop Dis. 2022 Feb 7;16(2):e0009585. doi: 10.1371/journal.pntd.0009585 (PMC8865650; doi:10.1371/journal.pntd.0009585)
Supplement: S1 Table — (PDF) [file pntd.0009585.s001.pdf]

Gene	Forward primer (restriction enzyme)	Reverse primer (restriction enzyme)
For bacterial expression by *E. coli* Rosetta (DE3) pLacI		
TconTS1a	GCAAGCTTCAGTGCTGCGACCACATG (*Hind*III)	CGGGATCCGTCGCTCCCAGGCACACG (*Bam*HI)
TconTS2	GCAAGCTTGCCCAGTGCATCTCAACG (*Hind*III)	GCGGATCCAGACACGGGATGCACATC (*Bam*HI)
TconTS2-LD	GCAAGCTTCTGGAGGATGAGATGGAGG (*Hind*III)	
TconTS3	GCAAGCTTTCTGGAAACGGACGAACG (*Hind*III)	GCGGATCCGAGGTAAAGTGACTCCAGTTC (*Bam*HI)
TconTS3-LD	GCAAGCTTCTAGAAGACGAGCTGGAAAGC (*Hind*III)	
TconTS4	GCGTCGACATCCTACAAGAAAGCTC (*Sal*I)	GCGGATCCCCTTGCTGCTCTTTTAAGTAAT (*Bam*HI)
TconTS4-LD	GCGTCGACCTCGCTGACGAACTGAAG (*Sal*I)	
Epitope mapping for anti-TconTS mAb 7/23		
TconTS1a-CD (1)	GCAAGCTTCAGTGCTGCGACCACATG (*Hind*III)	CGGGATCCGTCACTCGATTCGAATATC (*Bam*HI)
TconTS1a-CD (2)		CGGGATCCATCCGAGCTGCCAGGACCA (*Bam*HI)
TconTS1a-CD (3)		CGGGATCCATCACGATAATACGAGCCCT (*Bam*HI)
TconTS1a-CD (4)		CGGGATCCGTCCTGTGCCTTCCACACC (*Bam*HI)

TconTS1a-CD (5)		CGGGATCCGTCCACAAGGCGCA CAAGG (*Bam*HI)
TconTS1a-LD (6)	GCAAGCTTGACGAGCTGAAAAGC (*Hind*III)	CGGGATCCGTCGCTCCCAGGCA CACG (*Bam*HI)
TconTS1a-LD (7)	GCAAGCTTAACTGCCTCCCGGGC (*Hind*III)	
*Eco*105I insertion for TconTS domain swap		
TconTS1a*-CD (*Eco*105I)	GCAAGCTTCAGTGCTGCGACCAC ATG (*Hind*III)	GCTACGTAATCGCCCGGGAGGC (*Eco*105I)
TconTS1a*-LD (*Eco*105I)	GCTACGTAAAATATGATCCCGGG (*Eco*105I)	CGGGATCCGTCGCTCCCAGGCA CACG (*Bam*HI)
TconTS2*-CD (*Eco*105I)	GCAAGCTTGCCCAGTGCATCTCA ACG (*Hind*III)	GCTACGTATTTGTTCAGTTGAC (*Eco*105I)
TconTS2*-LD (*Eco*105I)	GCTACGTAAAGCGCCGGAGCGG C (*Eco*105I)	GCGGATCCAGACACGGGATGCA CATC (*Bam*HI)
TconTS3*-CD (*Eco*105I)	GCAAGCTTTCTGGAAACGGACGA ACG (*Hind*III)	GCTACGTAACCATCCGGTGAGG (*Eco*105I)
TconTS3*-LD (*Eco*105I)	GCTACGTAGATTATACTGAGGG (*Eco*105I)	GCGGATCCGAGGTAAAGTGACTC CAGTTC (*Bam*HI)
TconTS4*-CD (*Eco*105I)	GCGTCGACATCCTACAAGAAAGC TC (*Sal*I)	GCTACGTACCCGGTAGTTGCAG (*Eco*105I)
TconTS4*-LD (*Eco*105I)	GCTACGTAGATGGCAGCGATTGC (*Eco*105I)	GCGGATCCCCTTGCTGCTCTTTT AAGTAAT (*Bam*HI)

*Mutated TconTS containing the inserted *Eco*105I restriction site. Endonuclease restriction sites are underlined.
