# Supplementary material for: Cooperativity of catalytic and lectin-like domain of Trypanosoma congolense trans-sialidase modulates its catalytic activity
Source: PLoS Negl Trop Dis. 2022 Feb 7;16(2):e0009585. doi: 10.1371/journal.pntd.0009585 (PMC8865650; doi:10.1371/journal.pntd.0009585)
Supplement: S2 Table — (PDF) [file pntd.0009585.s002.pdf]

Enzyme	Trans-sialidase activity<br>Amount **3'SL** (pmol/min/ng TS)	Hydrolytic release of free Neu5Ac<br>Amount **Neu5Ac** (pmol/min/ng TS)	Transfer efficiency
**Expressed in *E. coli* Rosetta pLacI**			
TconTS1a	$2.79 \times 10^{-3} \pm 4.7 \times 10^{-5}$	$1.19 \times 10^{-4} \pm 1.5 \times 10^{-5}$	23
TconTS1*	$2.81 \times 10^{-3} \pm 4.1 \times 10^{-5}$	$1.17 \times 10^{-4} \pm 8.2 \times 10^{-6}$	24
TconTS1a/TS3	$8.62 \times 10^{-5} \pm 4.4 \times 10^{-6}$	$1.57 \times 10^{-4} \pm 1.6 \times 10^{-5}$	0.5
TconTS3	$4.15 \times 10^{-5} \pm 9.4 \times 10^{-6}$	$0.81 \times 10^{-4} \pm 1.2 \times 10^{-5}$	0.5

Quantifications of reaction products 3'SL and released free Neu5Ac were done employing HPAEC-PAD analysis as described under Methods. Transfer efficiency is defined as the ratio of 3'SL over Neu5Ac in the presence of the acceptor substrate lactose. Data points are means of triplicates ± standard deviation. *Mutated TconTS containing the inserted *Eco*105I endonuclease restriction site. Enzymes were incubated for 30 min at 37°C as described under Methods.
