# Supplementary figures and images for: Cooperativity of catalytic and lectin-like domain of Trypanosoma congolense trans-sialidase modulates its catalytic activity
Source: PLoS Negl Trop Dis. 2022 Feb 7;16(2):e0009585. doi: 10.1371/journal.pntd.0009585 (PMC8865650; doi:10.1371/journal.pntd.0009585)

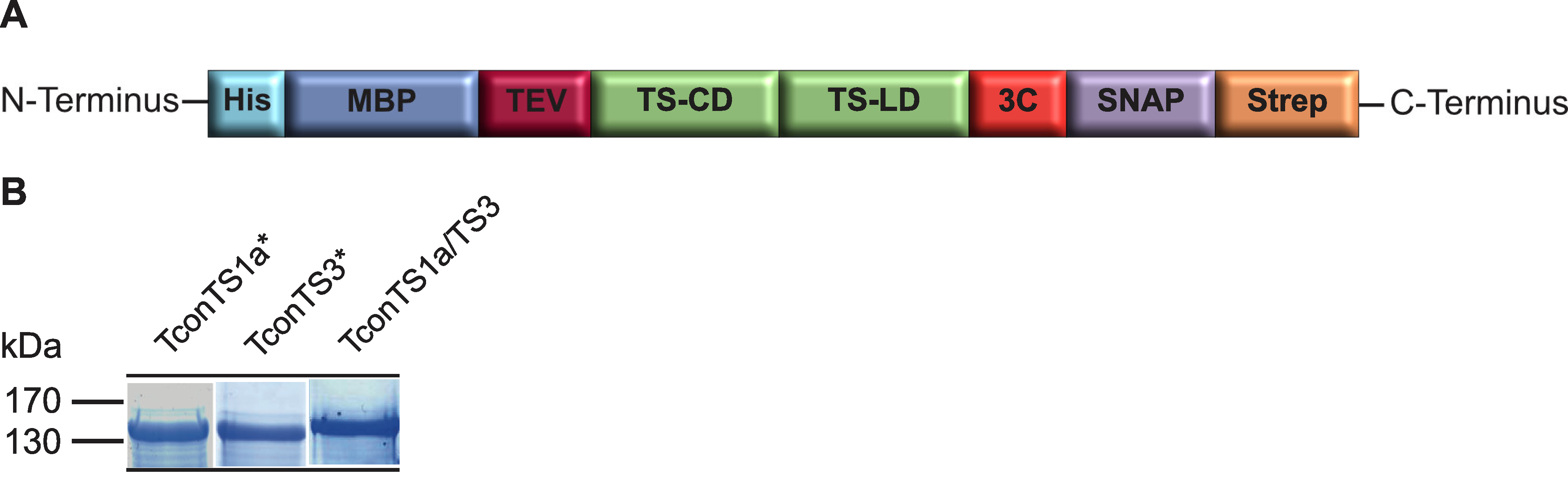

Supplement: S1 Fig — A: Schematic presentation of recombinant TconTS construct for expression in E.coli Rosetta pLacI. Fusion tags flanking TconTS are: MBP: maltose binding protein tag, TEV: tobacco etch virus protease cleavage site, 3C: human rhinovirus 3C protease cleavage site, SNAP: SNAP-tag, Strep: Strep-tag. B: SDS-PAGE of purified TconTS constructs. 1–2 μg of protein were loaded as indicated on a 10% SDS polyacrylamide gel, which was stained with Coomassie Brilliant Blue after electrophoresis. TconTS constructs with Eco105I restriction site inserted are indicated by *. Lane 3 comprise the domain swapped construct TconTS1a/TS3. (TIF) [file pntd.0009585.s003.tif]

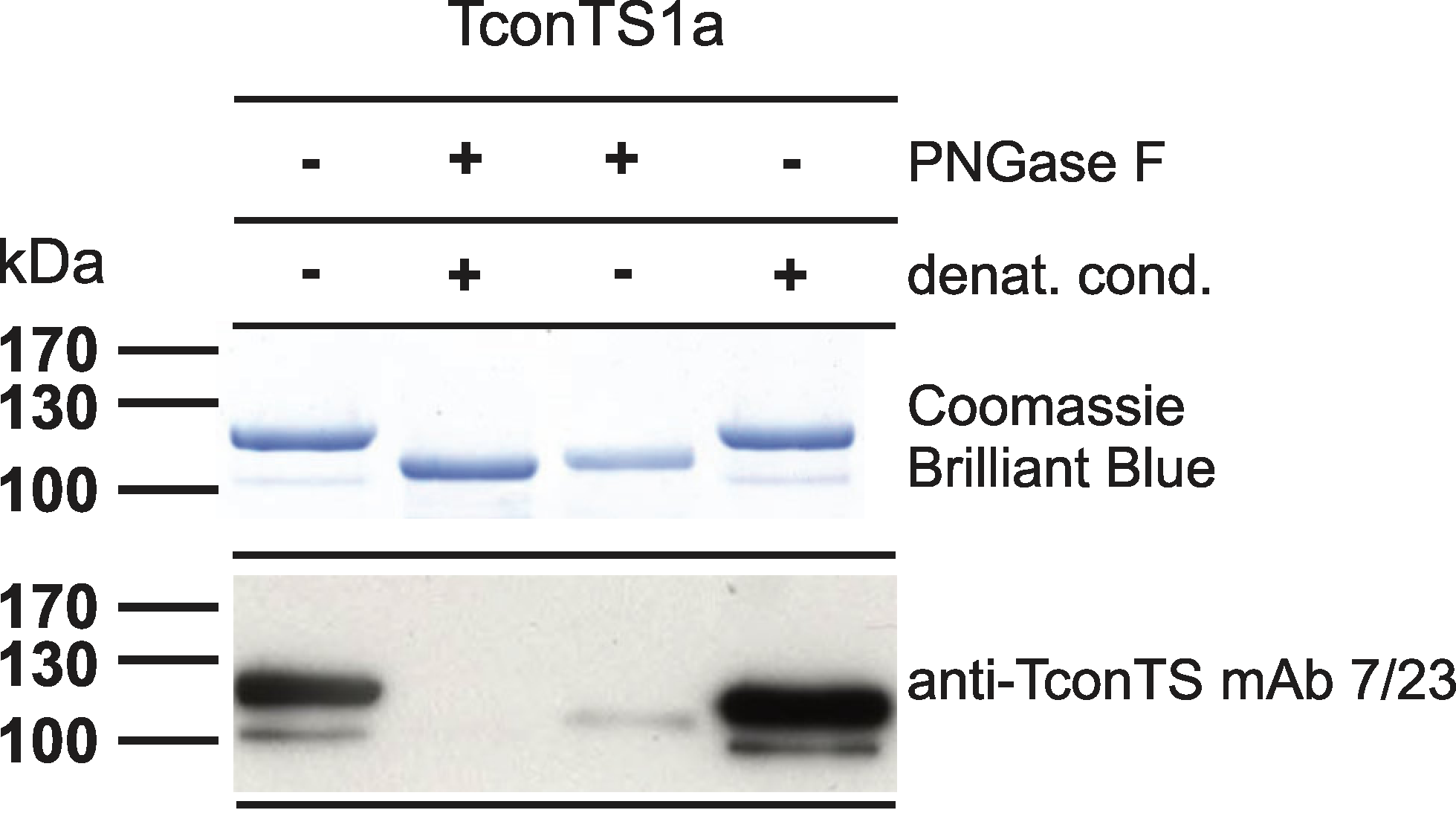

Supplement: S2 Fig — CHO-Lec1 expressed recombinant TconTS1a was deglycosylated using PNGaseF under native or denaturing conditions (denat. cond.) as described under Methods. 1 μg of TconTS1a was used in SDS-PAGE analysis with subsequent Coomassie Brilliant Blue staining and 100 ng in Western blots using anti-TconTS mAb 7/23 and anti-Strep-tag for detection as indicated (details under Methods). (TIF) [file pntd.0009585.s004.tif]

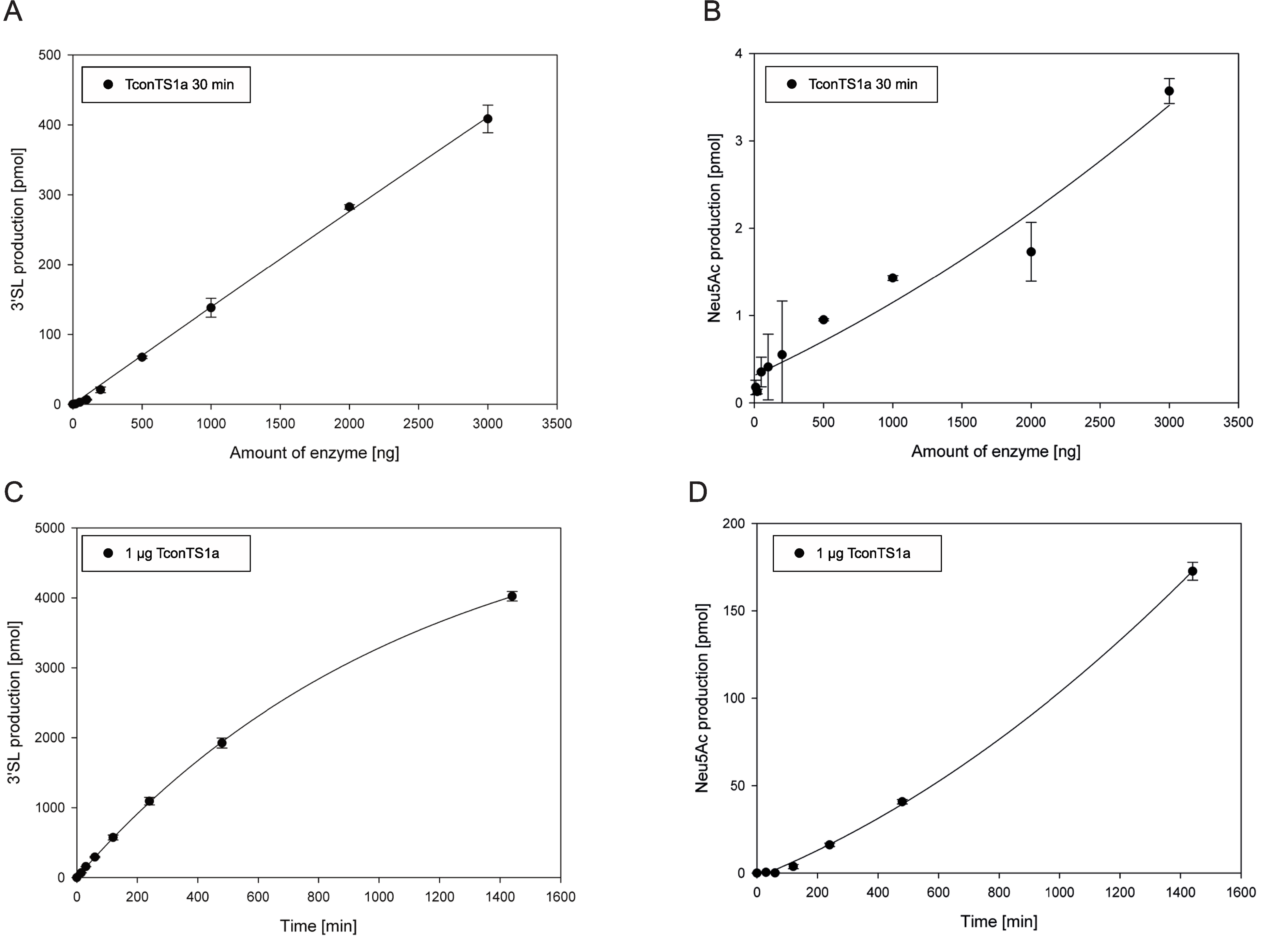

Supplement: S3 Fig — A-B: TconTS1a concentration dependent production of 3’SL (A) and hydrolytic release of Neu5Ac (B) using up to 1 μg of purified, bacterial expressed, recombinant TconTS. TS reactions were set up and analysed as described under Methods. Standard conditions with 100 μg fetuin (600 μM fetuin-bound Sia) and 2 mM lactose as Sia donor and acceptor substrates were incubated for 30 min at 37°C. C-D: Time dependency of 3’SL production and hydrolytic release of Neu5Ac. Reactions were incubated for the indicated times ranging from 0–1440 min with 1 μg of purified TconTS1a with standard fetuin and lactose concentrations (see Methods). Data points are means ± standard deviation of triplicates. (TIF) [file pntd.0009585.s005.tif]
