# Supplementary material for: Cooperativity of catalytic and lectin-like domain of Trypanosoma congolense trans-sialidase modulates its catalytic activity
Source: PLoS Negl Trop Dis. 2022 Feb 7;16(2):e0009585. doi: 10.1371/journal.pntd.0009585 (PMC8865650; doi:10.1371/journal.pntd.0009585)
Supplement: Attachment — Submitted filename: Response_to_Reviewers_Comments_PNTD-D-21-01140.docx [file pntd.0009585.s008.docx]

**Response to review comments**

Manuscript: *Cooperativity of catalytic and lectin-like domain of T. congolense trans-sialidase modulates its catalytic activity* (PNTD-D-21-01140)

We would like to thank the editorial board of PLOS Neglected Tropical Diseases for the opportunity to revise our manuscript and all reviewers for their critical reading and constructive comments on the various points. After reading the reviewers response we have realised that our study, as we originally submitted it, had several misunderstandings and logical breaks. After an intensive, critical examination of the comments, we have made the focus of our manuscript more stringent or realigned it accordingly, taking the reviewers' comments into account. As suggested by the reviewers, some passages in the manuscript have been changed, moved or presented as supplemental data in the revised version.

Our detailed response to the comments of all reviewers is presented below. Reviewer’s comments/questions are marked in blue and our corresponding reply in green. As it can be seen, in some cases we have subdivided the comments and questions into separate parts in order to be able to answer them more specifically. As offered by the editorial board of PLOS Neglected Tropical Diseases, we are very pleased to get the opportunity to resubmit our significantly revised manuscript "Cooperativity of catalytic and lectin-like domain of *T. congolense* trans-sialidase modulates its catalytic activity".

Therefore we have prepared two versions as stated by the editorial board.

1. Clean version (Revised manuscript)
2. Version with changes included (Changes are marked in red)

Reviewer response

**Methods:**
-Are the objectives of the study clearly articulated with a clear testable hypothesis stated?
-Is the study design appropriate to address the stated objectives?
-Is the population clearly described and appropriate for the hypothesis being tested?
-Is the sample size sufficient to ensure adequate power to address the hypothesis being tested?
-Were correct statistical analysis used to support conclusions?
-Are there concerns about ethical or regulatory requirements being met?

Reviewer #1: The structure of this article is rather confusing. Authors analyze the impact of the LD domain of TconTSs on the enzymatic activity. After a detailed analysis through modelling several TSs originating from different organisms, they reach the already known conclusion that CD and LD domains are held together through non-covalent unions, which defines a large area of interaction. As they hypothesize that LD domains influence the enzymatic activity, they decide to exchange the LD domain between several different TSs. However, they only focus in one construction between two TconTSs (so, why the structural analysis of so many TSs and on all the TconTSs?)

Reply 1: We thank the reviewer for the comment. We performed such a detailed computational analysis to identify common features of molecular interactions present at the interface between CD and LD. On this basis we calculated domain swapped TconTS1a/TS3 as a proof-of-principle for the recombination on an atomistic level. We added a short paragraph in line 556 of the revised manuscript. Obtained data provide the structural basis for the potential fusing CD of TconTS1a and LD of TconTS3 *in vitro* and resulting in an enzymatically active domain swapped TconTS1a/TS3.

In our study we have focused on TconTS1a/TS3 as a model system and proof-of-principle for the exchange of CDs and LDs of TconTS. Our study addresses the activity of TconTS1a and not all TconTS, which was initially misleadingly presented by us. We have made this point more clear in the abstract (line 37 and 48, revised manuscript) and adjusted the last paragraph of the introduction starting from line 182 (revised manuscript) accordingly. In addition, we have condensed Table 4 and removed TconTS constructs which were not investigated in this study

Reviewer #2: See general comments

Reviewer #3: The objectives of the study are cleary presented and from a methodological point of view, the study is well conducted. However, the authors fall short on the sample size (no. of TS swaps) to adequately address the aims of the study: to understand the effect of the LD-domain on TS activity. Statistical analysis support their conclusions and there are no specific ethical of regulatory requirements involved.

Reply 2: We thank the reviewer for the comment. In this study we only focus on the swap construct TconTS1a/TS3 as a model system and proof-of-principle for domain swapping CDs and LDs of TconTS and asked the question of whether TconTS3-LD modulates enzymatic activity of TconTS1a-CD (also see Reply 1). We did not intend to give the impression that we will provide a statement for all possible swap constructs with this study. We do agree that a detailed elucidation of underlying molecular mechanisms represent an important and interesting aim but these aspects will be part of a follow up study. In this study we provide the proof-of-principle that TconTS3-LD influences the activity of TconTS1a-CD.

We have made this point more stringent in the manuscript and changed the corresponding text passages accordingly (Line 48 (Abstract), line 182 and 252 (Introduction), line 558, (Results), line 990, 1291, 1407 (Discussion)).

**Results**:
-Does the analysis presented match the analysis plan?
-Are the results clearly and completely presented?
-Are the figures (Tables, Images) of sufficient quality for clarity?

Reviewer #1: Authors discard the use of the alpha helix that links the CD with the LD as swapping target, as it seems structural. They instead decide to use a Cys-flanked loop known to be variable and even disposable for enzyme activity. A decision that was experimentally proven as the correct one. Authors then proceed to detail all “possible” constructs in Table 4, but make and test only one (TS1/TS3). They state (line 527) “The crucial question of this study was which impact replacing the LD of TconTS1 with that from TconTS3 would have on the enzymatic activity of the fusion protein TconTS1a/TS3”, so why mention all the variants?

Reply 3: We thank the reviewer for the critical comment. We do recognize the confusion caused and agree to constrain the table. We have removed all domain swapped constructs except TconTS1a*, TconTS3* and TconTS1TS3 from Table 4 and from the rest of the manuscript accordingly.

And why include TS2 and TS4 in Fig 4C? Why are the authors not focusing on the only experimental chimeric molecules they generated? It is very confusing. In addition, the legend corresponding to Fig 4C, mentions a non-existent lane 6.

Reply 4: We thank the reviewer for the comments. We have removed TconTS2 and TconTS4 from Fig 4C and corrected the corresponding figure legend accordingly. He have corrected the description of the lanes in Fig 4.

Fig 4E shows that the glycosylation sites are conserved following the Eco105I restriction site insertion, a completely expected result as it inserts a YV dipeptide without disrupting any glycosylation consensus sequences. Besides, glycosylation is later demonstrated by PNGase treatment in Fig 5.
The major concern is the determination of the TS/sialidase activities themselves. The sialidase activity should be properly determined in the absence of an acceptor sugar as these (and other) authors have routinely done. This will measure its ability to hydrolyze the sugar, thus providing an accurate determination of the sialidase activity, which will then allow for comparative analysis with other reports. Testing free Sia with standard donor-acceptor molecules (specially under saturating conditions) does not accurately determine sialidase activity. The absence of determination of sialidase activities by MuNANA or fetuin  hydrolysis in the absence of acceptor suggars, as the authors usually do, is one of the main flaws of this report.

Reply 5: We thank the reviewer for the critical comment. We do agree that our initial presentation is misleading. Sialidase activity is not the right term in this context, for reasons that have already been very well described within the comment. Therefore, TS/sialidase activity is also misleading to describe and compare the enzymatic activity of TconTS constructs determined in this study. In this study we actually aim to investigate the influence of TconTS3-LD on Sia transfer efficiency of TconTS1a-CD by swapping domains. In order to determine the Sia transfer efficiency of TconTS1a/TS3, we quantified the transfer product 3’SL and hydrolytic side product Neu5Ac. We were then interested in how much hydrolytic side activity can be observed and thus how efficient is the Sia transfer for TconTS1a/TS3 relative to TconTS1a? This Sia transfer efficiency can only be determined in the presence of an suitable acceptor substrate such as lactose. We have pointed this out more clearly in the manuscript and added corresponding sentences in the abstract (line 37, 42-46), introduction (line 128, 144 and 152), results (line 519-526, 538, 541, 563-568) and the discussion section (line 580, 584-587, 673, 705, 716, 720 and 755) accordingly. We also adjusted Table 5 by changing “sialidase activity” to “hydrolytic release of free Neu5Ac” and “TS/ sialidase” to “Transfer efficiency”.

Line 447: …for TconTS3 no sialidase activity could be detected under conditions used [23].
However, ref 23 conditions are different as authors did not use acceptor molecules there, and thus the results obtained here are not comparable.

Reply 6: We thank the reviewer for the comment. We agree that sialidase activities is misleading in our study. However, the reason for our choice to fuse TconTS1a-CD and TconTS3-LD as claimed in Line 447 (initial version of the manuscript), is related to the differences in Sia transfer efficiencies in presence of acceptor substrate (Gbem 2013 PhD Thesis: http://nbn-resolving.de/urn:nbn:de:gbv:46-00103536-16; Table 4 and Table 5, page 78-79:).

It seems like TconTS3 does not release Neu5Ac from serum glycoproteins such as Fetuin in the presence of an acceptor substrate such as lactose. We have reorganized and adjusted a corresponding paragraph starting from line 128 (revised manuscript) and added the additional reference (Gbem 2013 PhD Thesis) accordingly. In addition, we have adjusted the sentence in line 436 (revised manuscript).

Line 553: ….after 120 min of incubation indicates that pTconTS1a does not release free Neu5Ac from fetuin but rather from the reaction product 3’SL.
It seems authors consider the Sia transfer occurs in strictly unidirectionaly from fetuin to lactose, probably due to the starting concentrations (600uM vs. 2mM). However, once 3’SL is generated, it becomes a Sia donor not only in hydrolysis but also transferences and can even back regenerate fetuin. Is there any known substrate preference in these enzymes?

Reply 7: We thank the reviewer for the comment. Under selected standard TconTS reaction conditions (600 µg fetuin bound Sia and 2 mM lactose) we do observe a preference towards Sia transfer from fetuin to lactose due to the high amounts of acceptor substrate. In our previous study we investigated, that in the competing activity using 3’SL as donor substrate for resialylation of asioalofetuin (ASF), free Sia is detected very early in the reaction (Koliwer-Brandl et al. 2011). Therefore we suggest that the free Sia detected in TS reactions (120 min) with fetuin as donor substrate is mainly the product of a hydrolytic side activity of the reaction using 3’SL as substrate. This assumption is further supported by the fact that lower amounts of TS can led to the same amount of final 3’SL but produce less free Neu5Ac as side product (Koliwer-Brandl et al. 2011). In addition we have also demonstrated that TconTS1 are able to transfer Sia from 3’SL to glycoproteins (Koliwer-Brandl 2011). However, the tendency of TconTS1 to release free Neu5Ac under standard conditions is more pronounced when using 3’SL and ASF as Sia donor and acceptor substrates.

As pointed out by several comments of the reviewer we decided to shift the data on enzymatic activity of bacterial expressed TconTS to supplement material (S1 Figure, S3 Figure and S2 Table) due to conceptional and technical reasons and to prevent misleading interpretations. We also exclude activity data obtained from bacterial expressed TconTS for the discussion regarding the influence of TconTS3-LD on Sia transfer efficiency of TconTS1a-CD. The reason for this decision is the lower overall enzymatic activity observed for TconTS expressed in bacteria most likely due to improper folding as indicated by the relatively high amount of insoluble TconTS. CHO-Lec1 cells represent a more suitable expression system for TconTS constructs then *E. coli* in order to answer our main research questions.

We have added a paragraph starting from line 666-675 (revised manuscript) that focuses on this subject accordingly.

Line 554: "Along this line, when considering the TS over sialidase activity (TS/sialidase) ratio pTconTS1a shows clear preference towards Sia-transfer to lactose (Table 5)."
Sialidase activity measured as free Sia in the presence of acceptor sugars is not comparable with previous references (see above).

Reply 8: We thank the reviewer for the comment. We have adjusted Table 5 accordingly. We have changed “sialidase activity” to “ hydrolytic release of free Neu5Ac” and “TS/sialidase” to “Transfer efficiency” due to reasons described in Reply 5. In addition, we have added the corresponding reference Gbem 2013 (dissertation) to the table footnotes accordingly.

In Table 5 authors seem to use Neu5Ac as equivalent to Sia. However, in addition to Neu5Ac, bovine fetuin also contains glycoyl sialic. Does these enzymes show any substrate preference?

Reply 9: We thank the reviewer for the comment. It has been found that the efficiency of TconTS to transfer Neu5Ac and Neu5Gc to acceptor substrates is same (Engstler et al., 1995). In addition to that, our HPAEC-PAD measurements did not show any peak for hydroxylated sialic acid product (Neu5Gc) when using fetuin as Sia donor substrate. Most likely because the content of Neu5Gc on bovine fetuin is known to be less then 3 % (overall content of fetuin-bound sialic acid).

Reviewer #2: See general comments

Reviewer #3: Trans-sialidase (TS) is an important enzyme produced by Trypanosomatids. As pointed out by the authors, due to their exclusive activity in these organisms, these enzymes are a pharmacological target of interest for the development of anti-trypanosmatid agents. 
TS enzymes contain two domains, the sialidase catalytic domain (SD) and the LamG-like lectin-like domain (LD). Here, the authors performed extensive bioinformatic/structural studies to understand the interaction between the domains and how they could affect the overall activity of the enzyme. They measure this as the ratio of TS activity x sialidase activity, or failure to transfer the sialic acid to a suitable acceptor molecule. 
However, although they have presented some very interesting structural data, it seems to me that the study in its present form is still preliminary and lacks more in-depth analysis and experiments. Most importantly, their overall conclusion is based on a single fusion construct with contrasting data (bacterial x mammalian).

Reply 10: We thank the reviewer for the detailed comment. We do recognise that the focus of our initial manuscript was misleading. Our study focuses on the proof-of-principle for swapping CDs and LDs of different TconTS. In addition to that domain swapped TconTS1a/TS3 represents the model system demonstrating that TconTS3-LD enhances Sia transfer efficiency in TconTS1a/TS3 relative to TconTS1a (also see Reply 1 and 2).

We agree that additional experiments on domain-swapped TS will be interesting in order to study cooperativity of CD and LD in more detail on a molecular level. However, our study provides the proof-of-principle to swap domains of TconTS which has led to functional TconTS1a/TS3 enzyme. The performed enzymatic activity determination on domain-swapped TconTS1a/TS3 provide a first proof of concept and clear evidence for cooperative interactions of CD and LD of TconTS.

Along, this line, we have adjusted the manuscript and only focused on domain swapped TconTS1a/TS3 accordingly as stated in Reply 1 and 2.

In addition we have recognized that comparing enzymatic activity data for TconTS expressed by E. coli and CHO-Lec1 cells does not help to answer our main research question but rather tends to lead to misleading or false interpretations. Therefore, we have decided to move the enzymatic activity data on TconTS expressed by bacteria to supplement material and only focus on data obtained by CHO-Lec1 expressed enzymes as stated in Reply 7. We have stringent the manuscript in that direction accordingly.

Strong points: the authors have performed a very nice study to map the contact points between the SD and LD domain. 

Week points: based on their map, they chose to swap domains of two TS to generate a single swap construct (TconTS1a/TS3) which was produce in bacterial and mammalian cells. In bacteria, this swapped construct changed its activity profile from a more TS to a more SA enzyme activity. Nevertheless, the same construct when produce in mammalian cells display the opposite effect, with even more TS activity.

Reply 11: We thank the reviewer for the comment. We agree that comparing enzyme activity of TconTS from both *E. coli* and CHO-Lec1 expression systems is misleading and does not help in answering the research questions of our study. We have discussed this subject in Reply 7. Also we have adjusted the parameter “sialidase activity” to “hydrolytic release of free Neu5Ac” and “TS/sialidase ratio” to “transfer efficiency” as stated in Reply 5 accordingly.

It seems to me that it is difficult to reach any conclusion regarding the effect of domain combination in the TS family based on these results. The author would have to generate many more constructs (SD1/LD2 x SD2/LD1 compared their corresponding wt1 and wt2, etc... for at least 2 or 3 different TS enzymes).

Reply 12: We thank the reviewer for the comment. We do agree that our study does not provide data for the TS family in detail but has demonstrated the proof-of-principle for the cooperativity of CD and LD of TconTS1a-CD and TConTS3-LD. We have adapted the manuscript accordingly. Please also see Reply 1, 2 and 7.

The way I see this is: trypanosomatids contain hundreds of copies of TS genes that were generated by gene duplication during evolution. Nature probably selected combinations of SD/LD copies that worked well and eliminated those that worked poorly (natural swapped generated during evolution). So, it is not surprising that changes of the LD domain of a given TS gene would change the behavior of the enzyme. The question is: what can we learn from these different combination? What is their molecular base? Based on this study in its present form, not much, unfortunately.

Reply 13: We thank the reviewer for the critical comment. We recognise that the results of our study were not well presented in the initial version of the manuscript resulting in misleading interpretations and confusion. However our study focuses on the question whether and to what extend TconTS3-LD affects catalytic activity of TconTS1a and provide a clear proof-of-principle and biochemical insights. As stated in Reply 1 and 2 we have revised the manuscript accordingly.

We are also convinced that TconTS variants and combinations of CD and LD have evolved from natural selection processes. However, our study provides for the first time a “proof-of-principle” that evolutionary domain swaps are possible for TS, and that they have an effect on enzymatic activity. We adjusted the corresponding text passages starting from line 112 and in line 640.

In addition, this study together with our previous study (Waespy et al. 2015) demonstrating the ability of TconTS-LD to bind oligomannose-oligosaccharides and our current study (Rosenau et al. 2021 https://biorxiv.org/cgi/content/short/2021.12.13.472379v1) demonstrating the involvement of oligomannose N-glycans of TconTS on enzymatic activity provides new aspects of biochemical properties of TconTS and TS in general. We assume that these biochemical aspects of TconTS-LD represent important data in order to understand molecular mechanisms of TconTS activity and substrate specificity.

We have added a corresponding sentence in line 757 (revised manuscript).

Minor issues:

Table 4 seems dispensable. The authors showed all possible combinations and then generated a single one?

Reply 14: We thank the reviewer for the comment. We do agree that some of the content of Table 4 is not supporting the manuscript. We have adjusted the Table 4, which now illustrates only TconTS constructs characterized in this study. Please also see Reply 1 and 2.

Furthermore, the results regarding PNGaseF digestion to remove N-linked glycosylation is strange and it seems like a side project. Usually, N-glycosylation usually blocks access to antibodies (cryptic epitopes). In these cases, it seems to me that lack of glycosylation may simply affect domain folding or the display of the epitope on the nitrocellulose/PVDF membrane; a technical artifact. Further experiments would be necessary to address this conundrum.

Reply 15: We thank the reviewer for the interesting comment. In the first instance, we were also very surprised and skeptical regarding the possible involvement of an *N*-glycan on TconTS1 to be part of the epitope recognized by the anti-TconTS mAb 7/23. Our data clearly indicate that *N*-glycan structures of TconTS1a have an influence on the recognition by the anti-TconTS mAb 7/23 antibody. However, in frame of our current study regarding the involvement of *N*-glycosylation of TconTS on enzymatic activity (Rosenau et al. 2021) we performed circular dichroism analysis of *N*-glycosylated and deglycosylated TconTS1 and found that enzymatic removal of *N*-glycans did not led to any changes in the secondary structure or protein stability of TconTS1a.

However, in light of this comment we decided to reduce the data on *N*-glycosylation of TconTS in this manuscript as it might only result misleading. We shifted the data regarding PNGaseF treatment of TconTS to supplemental material and adjusted the corresponding paragraph starting from line 504-516 and line 681-696 (revised manuscript) accordingly.

Other authors have showed the the LamG-lectin like domain bind to other substrates as well (ie., cytokeratin - Teixeira et al., PLOS Negl Trop Dis, 2015). None of these studies were cited by the authors or discussed in terms of how it could affect TS activity (by bringing specific acceptor molecules close to the SD domain?).

Reply 16: We thank the reviewer for the constructive comment. Starting from Line 726 (revised manuscript), we have discussed the possible binding of TconTS CD and LD to multivalent ligands such as cell surface glycoconjugates. In the same paragraph, we have also given the example of glutamic acid/alanine-rich protein (GARP) binding to TconTS and consequently modulating enzymatic activity. These findings provide strong evidence for a cooperative interplay of TconTS CD and LD promoting enzymatic activity. A prominent example for such a dual function of domains represent *Vibrio cholerae* neuraminidase. This bacterial neuraminidase consist of three domains whereas one bears sialidase activity while the second one is a galactose-binding domain, bringing the neuraminidase close to its substrates.

We have added a corresponding paragraph starting from line 741 of the discussion section (revised manuscript).

**Summary and General Comments**:
Use  this section to provide overall comments, discuss strengths/weaknesses of the study, novelty, significance, general execution and scholarship. You may also include additional comments for the author, including concerns about dual publication, research ethics, or publication ethics. If requesting major revision, please articulate the new experiments that are needed.

Reviewer #1: The article is interesting to clarify the biochemical properties of the TconTSs. However, it seems not structurally well written making it very confusing. Readers expect more results early on when they analyze many different TSs and then the TconTS between them, but end up studying a single chimeric construct. I can accept this as a "proof of principle" that the LDs actually play a significant role on the enzymatic activity. However, the sialidase activity will be more accurately evaluated in the absence of acceptor sugars.

Reply 17: We thank the reviewer for the comment. We recognise that our initial manuscript contains structurally ambiguous passages and interpretations, which may bring the reader a false expectation of the results presented here.

We agree that our study represents a “proof-of-principle” and have adjusted the whole manuscript accordingly. Please also see Reply 1 and 2 for more details in that respect.

In addition we have focused on the domain swap TconTS1a/TS3 and removed other possible constructs from Table 4.

We agree that the term “sialidase activity” is not appropriate in this context. Please see Reply 5 in that context.

Reviewer #2: In this manuscript the authors expand their previous results that showed that lectin domain (LD) of Trypanosoma trans-sialidase (TSs-) binds to oligosaccharides and now suggested that this binding modulates the transglycosylation reaction reducing the transference to water. Such conclusion could suggest modulatory effects of the enzyme by its own glycosylation or when it recognize different substrates. To show this modulation, the authors initially used TS of Trypanosoma congolense and generate structural models which indicate possible interactions through hydrogen bounds between the catalytic domain (CD) and the LD. The models also allowed them to swap the LD domains between a T. congolense TS1 and TS3, this later showing less trans activity, and verify the influence of the LD on both reactions. Finally, they showed that addition of mannotriose to recombinant enzymes decrease the transfer to water. 

The results add some novel information about this possible regulatory mechanism and indicate that the LD domain is key for preventing the hydrolysis reactions. However, the manuscript is quite difficult to follow as it mix key findings with controls and system definitions. There are some issues that confounds the reader and require a better organization of the text. I will list some topics that could be modified and improved. 

Major points
1. The use of recombinants obtained in E. coli versus mammalian cells create several doubts and it is difficult to distinguish whether the differences are due to protein glycosylation and improper folding and possible aggregation and/or denaturation. I think the use of the two systems does not help to explain a role of the LD domain in the activity modulation as it could just help to fold the beta-barrel. It would be interesting to further characterize the aggregation state, alpha-helix content or other structural property in parallel.

Reply 18: We thank the reviewer for the comment. We have recognised that we have delivered the wrong approach and generated confusion in that context.

Our study does not aim to make a comprehensive comparison between the two expression systems regarding the influence of LD on enzymatic activities.

Our data on enzymatic activity of TconTS expressed by *E. coli* and CHO-Lec1 cells demonstrate that there are differences in overall enzymatic activity, which is most likely just due to the quality of the samples as also indicated by the reviewer. TconTS expressed by CHO-Lec1 cells showed higher overall enzymatic activities indicating a relatively high proportion of correctly folded protein in the sample. Therefore we decided to exclude activity data obtained from bacterial expressed TconTS1a/TS3 for the discussion regarding the influence of TconTS3-LD on enzymatic activity of TconTS1a-CD and shifted the data to supplemental part (S1 Figure, S3 Figure and S2 Table).

Please see Reply 7 in that context.

We have adjusted the corresponding paragraph in the discussion section starting from line 666-675 (revised manuscript) accordingly.

2. The results of the mAb specific for TScon1, which recognize only after glycosylation is quite complex. As it recognize the proteins after SDS-PAGE, it seems that it is not directed to conformational epitopes. Therefore, it does not help to reach the main conclusion and confounds the reader.

Reply 19: We thank the reviewer for the comment. We do agree that mapping the binding epitope of the anti-TconTS mAb 7/23 does not directly support the main conclusion of our study. We just used the anti-TconTS mAb 7/23 as a tool to characterise the swap construct TconTS1a/TS3 as it recognizes an epitope present in TconTS1a-LD but not in TconTS3-LD. We indicated this idea in the paragraph starting from line 473-477 (revised manuscript) accordingly.

We agree that our data regarding the involvement of *N*-glycan structures on the recognition by the anti-TconTS mAb 7/23 antibody is quite complex, difficult to understand and not supporting the main conclusion of our study. Therefore we decided to move these data to supplemental part (S2 Figure). We have reduced the deglycosylation data to a short paragraph starting from line 511-516 and line 681-696 (revised manuscript).

Please also see Reply 15 in that context.

3. It is unclear whether the glycosylation type (high mannose and complex) is related to the activity and if they act by intramolecular interactions. This also confounds the reader to understand the role of glycosylation versus the addition of exogenous oligosaccharides. 

Reply 20: We thank the reviewer for the comment. Since we expressed recombinant TconTS constructs in CHO-Lec1 cells we expect *N*-glycosylation of TconTS to be of high-mannose type *N*-glycans. We have confirmed this in frame of our current study (Rosenau et al. 2021 https://biorxiv.org/cgi/content/short/2021.12.13.472379v1). We have also demonstrated that *N*-glycans modulate TconTS1 enzymatic activity possibly via intramolecular interactions with amino acid residues present at the catalytic site. We added a sentence in line 693-696 of the discussion section (revised manuscript).

In our study we have clearly demonstrated that the addition of 1,4β-mannotriose suppressed the hydrolytic release of Neu5Ac of TconTS1a in the presence of the acceptor substrate lactose as stated in line 716 (revised manuscript). We propose that this effect is due to the occupation of the carbohydrate binding site in TconTS1a-LD since TconTS1a-CD showed no binding to oligomannose *N*-glycans (Waespy et al. 2015) as also stated in line 718 (revised manuscript).

We have added sentences regarding the idea of using 1,4β-mannotriose to alter enzymatic activity of TconTS1a in line 152 and in the paragraph starting from line 583-589.

However, our study does not aim to understand the role of *N*-glycosylation since our current study (Rosenau et al. 2021) has targeted this objective as stated above. We added a sentence in line 757 (revised manuscript).

4. The strict comparison of the TS versus hydrolysis activity could be better performed (see below).

Reply 21: We thank the reviewer for the comment. We agree that sialidase activity is not the right term in this context. We have changed “sialidase activity” to “ hydrolytic release of free Neu5Ac” and “TS/sialidase” to “Transfer efficiency” in the manuscript. Please also see Reply 5.

Specific points

-Line 39:  It is unclear what is TS3 in the abstract for those who are not familiar with TScon

Reply 22: We have adjusted the corresponding text passage in the abstract accordingly.

-Line 42: It is unclear the relation between the mannotriose and the swap experiment and the conclusion have no clear support on the previous sentence.

Reply 23: We have modified the abstract and added a corresponding sentence in line 44 (revised manuscript) accordingly.

-The last paragraph of the Introduction describes the results in an unusual way. I would suggest modifying it.

Reply 24: We have modified the paragraph and hope that it now meets the expectations.

-Line 168. Fig. 5 citation appears before other figures (check the order in the final version)

Reply 25: We have adjusted the order of figures accordingly.

-It would be interesting to have the plasmid sequences deposited or available.

Reply 26: We have added the plasmid DNA sequences of pDEF-Transin-TconTS1a(Eco105I)-SNAP-Strep and pET28a-His-MBP-TconTS1a(Eco105I)-SNAP-Strep as supplemental Figure S4 and Figure S5 respectively.

-Line 223. Fig. 4A citation appears before other figures (check the order in the final version)

Reply 27: We have adjusted the order of figures accordingly.

-Line 224, CHO are derived from ovary. I’m not sure if are typical fibroblasts.

Reply 28: We thank the reviewer for the comment. Indeed CHO cells are not typical fibroblast rather fibroblast-like. We have removed the term “fibroblast” to avoid misunderstandings.

-Line 436. The sentence appears unlinked the description of expression in bacteria. This part of the text should be reorganized.

Reply 29: We have reorganized the whole paragraph and have revised the transition of the individual sections.

-Line 460, it should be stated that p is prokaryotic and e eukaryotic in the legend.

Reply 30: We have made the necessary change in the figure legend of Figure 4 accordingly.

-Line 524, it would be interesting to indicate the putative N-glycosylation sites in the protein.

Reply 31: We have performed such an analysis and presented the results in our previous study (Waespy et al. 2015). A sentence referring to these results is given in line 681 (revised manuscript).

-Line 541, the legend state that 3 ug of enzyme and in the figure is annotated 1 ug.

Reply 32: Of course it should be 1 µg of enzyme. We have corrected this typo accordingly.

-Line 552, the delayed release of free Neu5Ac could be due to a secondary release from SL formed after transfer from fetuin to lactose as stated. However, the results are still insufficient to proof this. A better kinetic analysis could be performed.

Reply 33: We have modified the corresponding sentence and added two references supporting these observations.

-Table 5. The time used for measurements should be stated. Are these initial velocities? The results are clearly different but to make statements and compare the ratio of TS/sialidase, it would be important to perform Michaelis determination (Km and Vms).

Reply 34: We thank the reviewer for the comment. We have added the reaction time in the figure legend accordingly. We recognise that “TS/sialidase activity” is misleading to describe and compare the enzymatic activity of TconTS constructs determined in this study. Therefore we changed the term “sialidase activity” to “hydrolytic release of Neu5Ac” and “TS/sialidase activity” to “Transfer efficiency” as this describes the activity more accurately. Focus of our study was to determine the Sia transfer efficiency of TconTS1a/TS3 relative to TconTS1a. Sia transfer efficiency can only be determined in the presence of an suitable acceptor substrate such as lactose.

In our study we focused on the efficiency of Sia transfer and not the isolated sialidase activity.

We have adjusted the manuscript at the corresponding text passages accordingly. Please see Reply 5 in this context.

- Table 5. The difference in activity could be a consequence of proper folding due to glycosylation, membrane export, cysteine bridge formation and glycosylation. But part of the prokaryotic enzyme could be denatured or aggregated. Further purification could be necessary. This could reflect the low production of protein in E. coli and their low specific activity when compared to the enzymes produced in CHO cells. I noticed that this is discussed but to be published some additional evidences should be provided. Also, it is important to describe how the proteins are stored.

Reply 35: We thank the reviewer for this comment. We agree that the expression of proteins of prokaryotic origin in *E. coli* is often accompanied with various problems (as already listed in the comment). In fact, we did not go into the details of this problem in this study, but concentrated on the CHO-Lec1 expressed enzymes. However, it has become clear that CHO-Lec1 cells represent a more ideal expression system for TconTS than *E. coli*. As also pointed out in the comment, we decided to shift the data on enzymatic activity of bacterial expressed TconTS to supplement material (S1 Figure, S3 Figure and S2 Table). A paragraph addressing this subject is added to the manuscript starting from line 660-675. Please also see Reply 7 in this context.

- Line 600. Is 5 mM of mannotriose compatible with its affinity. Perhaps, it would be important to titrate the effect to measure binding.

Reply 36: We thank the reviewer for the comment. We agree that titrating the ligand mannotriose would be an interesting objective but we think that more complex glycans would possibly lead to stronger effects on enzymatic activities, since we have identified these as potent competitive inhibitors in our previous study (Waespy et al. 2015). However, we feel that such experiments are beyond the scope of this manuscript with its key conclusion “proof-of-principle”.

Reviewer #3: (No Response)
